# Interaction of *Medicago truncatula* Lysin Motif Receptor-Like Kinases, NFP and LYK3, Produced in *Nicotiana benthamiana* Induces Defence-Like Responses

**DOI:** 10.1371/journal.pone.0065055

**Published:** 2013-06-04

**Authors:** Anna Pietraszewska-Bogiel, Benoit Lefebvre, Maria A. Koini, Dörte Klaus-Heisen, Frank L. W. Takken, René Geurts, Julie V. Cullimore, Theodorus W.J. Gadella

**Affiliations:** 1 Section of Molecular Cytology, Swammerdam Institute for Life Sciences, University of Amsterdam, Amsterdam, The Netherlands; 2 INRA, Laboratoire des Interactions Plantes-Microorganismes (LIPM), UMR441, F-31326 Castanet-Tolosan, France; 3 CNRS, Laboratoire des Interactions Plantes-Microorganismes (LIPM), UMR2594, F-31326 Castanet-Tolosan, France; 4 Section of Plant Pathology, Swammerdam Institute for Life Sciences, University of Amsterdam, Amsterdam, The Netherlands; 5 Department of Plant Science, Laboratory of Molecular Biology, Wageningen University, Wageningen, The Netherlands; University of North Dakota, United States of America

## Abstract

Receptor(-like) kinases with Lysin Motif (LysM) domains in their extracellular region play crucial roles during plant interactions with microorganisms; e.g. *Arabidopsis thaliana* CERK1 activates innate immunity upon perception of fungal chitin/chitooligosaccharides, whereas *Medicago truncatula* NFP and LYK3 mediate signalling upon perception of bacterial lipo-chitooligosaccharides, termed Nod factors, during the establishment of mutualism with nitrogen-fixing rhizobia. However, little is still known about the exact activation and signalling mechanisms of MtNFP and MtLYK3. We aimed at investigating putative molecular interactions of MtNFP and MtLYK3 produced in *Nicotiana benthamiana*. Surprisingly, heterologous co-production of these proteins resulted in an induction of defence-like responses, which included defence-related gene expression, accumulation of phenolic compounds, and cell death. Similar defence-like responses were observed upon production of AtCERK1 in *N. benthamiana* leaves. Production of either MtNFP or MtLYK3 alone or their co-production with other unrelated receptor(-like) kinases did not induce cell death in *N. benthamiana*, indicating that a functional interaction between these LysM receptor-like kinases is required for triggering this response. Importantly, structure-function studies revealed that the MtNFP intracellular region, specific features of the MtLYK3 intracellular region (including several putative phosphorylation sites), and MtLYK3 and AtCERK1 kinase activity were indispensable for cell death induction, thereby mimicking the structural requirements of nodulation or chitin-induced signalling. The observed similarity of *N. benthamiana* response to MtNFP and MtLYK3 co-production and AtCERK1 production suggests the existence of parallels between Nod factor-induced and chitin-induced signalling mediated by the respective LysM receptor(-like) kinases. Notably, the conserved structural requirements for MtNFP and MtLYK3 biological activity in *M. truncatula* (nodulation) and in *N. benthamiana* (cell death induction) indicates the relevance of the latter system for studies on these, and potentially other symbiotic LysM receptor-like kinases.

## Introduction

Legumes can establish a mutualism with compatible rhizobia ultimately leading to nodulation, i.e. a formation of specialized symbiotic organs (nodules) in which atmospheric dinitrogen is converted into ammonia by the bacteria in exchange for plant carbohydrates. Nod factors (NFs) play a central role during most *Rhizobium-*legume (RL) symbioses [1]. They are secreted rhizobial signals whose perception by host legume roots is required for root nodule organogenesis, invasion of rhizobia toward a nodule primordium, and accommodation of bacteria inside nodule cells [2]–[4]. In two model legumes, NF-induced responses during the pre-infection step of RL interaction require *M. truncatula* (*Medicago*) *Nod Factor Perception* (*MtNFP*), and *Lotus japonicus* (*Lotus*) *Nod Factor Receptor 1* and *5* (*LjNFR1* and *LjNFR5*) [5]–[11]. At a later step, *LjNFR1*, *LjNFR5*, *MtNFP*, and an additional *Medicago* gene, *LysM domain-containing Receptor-Like Kinase*/*Root Hair Curling* (*MtLYK3*/*MtHCL*), are required for rhizobial infection via so-called infection threads through which the bacteria penetrate nodule primordia [9], [12]–[15]. Additionally, *MtNFP* and *MtLYK3* might co-function during nodule development and/or accommodation of rhizobia inside nodule cells [9], [16]–[18]. Recently demonstrated binding of NF derivatives to LjNFR1 and LjNFR5 confirmed their role as NF receptors [19], whereas the exact mechanism of MtNFP and MtLYK3 activation by compatible NFs remains to be shown.

All four genes encode receptor-like kinases (RLKs) with an extracellular region (ExR) predicted to contain three LysM domains, a transmembrane helix, and a protein kinase domain (KD) within the intracellular region (InR) [5], [20]–[22]. Remarkably, in contrast to MtLYK3 and LjNFR1, which both display kinase activity, MtNFP and LjNFR5 seem to function as pseudokinases that neither show nor rely on the intrinsic kinase activity to signal [9], [20]–[22]. LjNFR5 is hypothesized to form a receptor complex with LjNFR1: a notion consistent with their demonstrated co-functioning during the determination of RL specificity [23]. Similarly, a receptor complex composed of MtNFP and a yet-unidentified LysM-RLK or MtLYK3 is predicted to initiate the pre-infection responses and the infection process, respectively [12], [15]. Since mutagenesis studies in *Medicago* have not identified alterations in genes other than *MtNFP* that lead to complete lack of responsiveness to NFs, a function of this additional LysM-RLK in the pre-infection stage is most likely redundant. In addition, MtNFP has been implicated in *Medicago* interactions with pathogens (*Aphanomyces euteiches* and *Colletotrichum trifolii*), and with beneficial arbuscular mycorrhiza (AM) fungi [24]–[27]. However, it remains to be shown whether MtNFP functions in these processes alone or in co-operation with (an)other RLK(s).

LysM-RLKs in non-legume species also govern plant-microbe interactions. An *MtNFP*/*LjNFR5* homolog in *Parasponia andersonii*, *PaNFP*, is involved in interactions with *Sinorhizobium* sp. NGR234 and *Rhizophagus irregularis* (formerly *Glomus intraradices*), resulting in nitrogen-fixing and arbuscular mycorrhiza symbiosis, respectively [28]. *Arabidopsis thaliana* (*Arabidopsis*) *LysM-RLK1*/*CERK1* (*Chitin Elicitor Receptor Kinase 1*) and its ortholog from rice (*Oryza sativa*), OsCERK1, are essential for microbe-associated molecular pattern (MAMP)-triggered immunity. MAMPs are specific molecules conserved in various classes of microorganisms that activate receptor-mediated defence signalling [29]–[30]. CERK1-mediated innate immunity to fungal and bacterial pathogens is activated upon perception of chitin/chitooligosaccharides (COs), or peptidoglycan (PGN), respectively [31]–[35]. In the latter case, both in rice and in *Arabidopsis* PGN binds not to OsCERK1/AtCERK1 but to extracellular LysM domain-containing proteins, termed LYPs or LYMs [35]–[36]. This in turn is postulated to induce a formation of AtCERK1/AtLYMs receptor complexes, and subsequent signal transduction via the kinase activity of AtCERK1. A similar mechanism operates during COs-induced signalling in rice, involving OsCERK1 and (a) LYP protein(s) [34], [36], whereas in *Arabidopsis* COs bind directly to AtCERK1 [37]–[38]. Therefore, modes of CERK1 activation, even upon perception of the same MAMP, can differ between plant species.

We are interested in NF-induced signalling mediated by MtNFP and MtLYK3, focusing on their postulated interaction *in situ*. However, our attempts to visualize these proteins in *Medicago* root have been unsuccessful, presumably due to stringent regulation of their accumulation (even in the situation of an attempted overproduction). *Nicotiana benthamiana* (*Nicotiana*) has proved to be a useful model for heterologous production and structure-function studies on multiple proteins, providing invaluable insights that guided their subsequent analyses in the respective homologous systems [39]. Therefore, we employed an *Agrobacterium tumefaciens* (*Agrobacterium*)-mediated transient transformation of *Nicotiana* leaves [40], which allowed us to produce both proteins to levels suitable for fluorescence microscopy. Remarkably, we found that heterologous co-production of MtNFP and MtLYK3 resulted in the induction of defence-like responses that are typically observed upon treatment with pathogen-derived molecules. As the apparent (functional) interaction of these LysM-RLKs in *Nicotiana* activated defence-like responses, similar to these mediated by AtCERK1, our results indicate the existence of parallels between NF-induced and COs-induced signalling.

## Materials and Methods

### Constructs for Plant Expression

The cDNA of *MtNFP*, *MtLYK3*, and *MtDMI2* (in the latter case the first intron was included in the cDNA), and the genomic sequence of *AtCERK1* were PCR amplified and cloned in a pMON999 vector containing a CaMV 35Sp:: (*sYFP2*, *mCherry*, *3xFLAG* or empty) 35S terminator cassette. The stop codon was removed from the coding sequences during cloning (except when generating untagged *MtNFP* and *MtLYK3* constructs) to allow a translational fusion. Sequences of the primers and linkers are given in Table S1. All point mutations were introduced as described [20]. All constructs were sequenced to verify the correct insert sequence. Constructs generated in pMON999 vector were subsequently recloned into a pBin+ (all *MtNFP* constructs) or pCambia1390 (all *MtLYK3*, *AtCERK1*, and *MtDMI2* constructs) vector using HindIII and SmaI sites. *MtNFP*[ΔInR], *MtNFP*-*YFP*_N_, *MtLYK3*-*YFP*_N_, *MtLRRII.1-YFP*_C_, *AtBRI1-YFP*_C_ (where *YFP_N_* or *YFP*_C_ encode, respectively, the N- and C-terminal part of split YFP used in BiFluorescence Complementation assay) constructs are described [22], [41].

### *Nicotiana* Transformations

**Figure 1:**
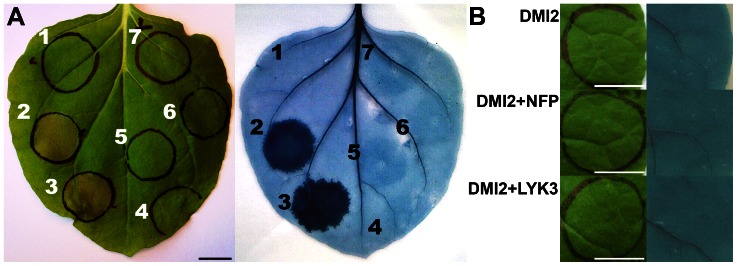
Co-production of MtNFP and MtLYK3 induces cell death in *Nicotiana* leaves. A, The following *MtNFP* and *MtLYK3* constructs were expressed alone or co-expressed in *Nicotiana* leaves: mock infiltration (1); *MtNFP* untagged+*MtLYK3* untagged (2); *MtNFP-sYFP2*+*MtLYK3-sYFP2* (3); *MtLYK3-sYFP2* (4); *MtLYK3* untagged (5); *MtNFP-sYFP2* (6); *MtNFP* untagged (7). Macroscopic observation (left panel) and subsequent Evans blue staining (right panel) are depicted 48 hai. Bar is 1 cm. B, *MtDMI2-sYFP2* construct was expressed alone or co-expressed with either *MtNFP-mCherry* or *MtLYK3-mCherry* construct in *Nicotiana* leaves. Macroscopic observations (left panel) and subsequent Evans blue stainings (right panel) are depicted 48 hai. Bars are 1 cm.

**Figure 2:**
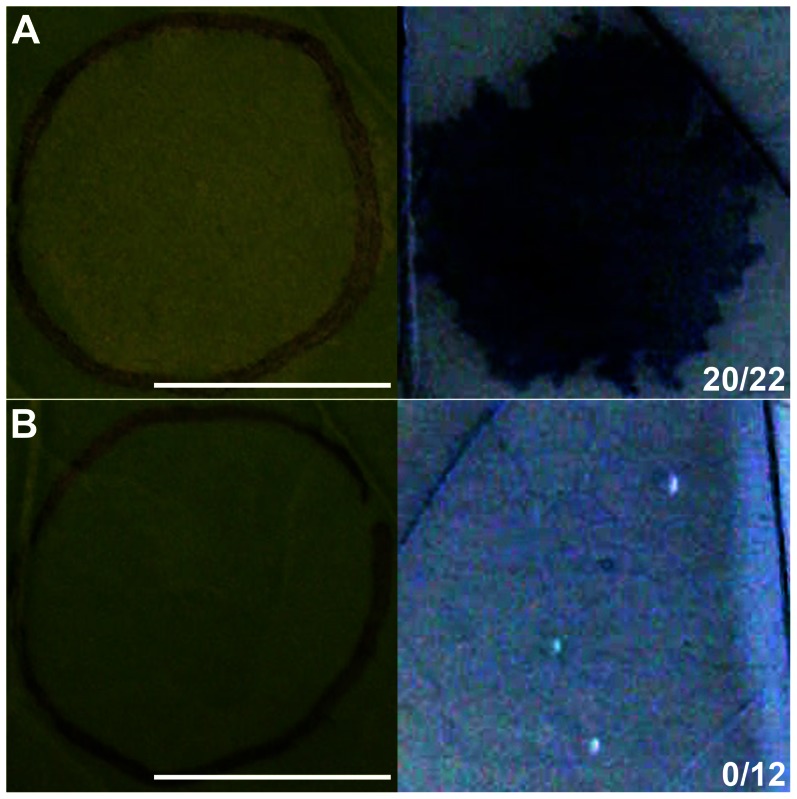
Production of AtCERK1 in *Nicotiana* leaves induces cell death that requires AtCERK1 kinase activity. *AtCERK1-sYFP2* (A) and *AtCERK1*[K349E]-*sYFP2* (B) constructs were expressed in *Nicotiana* leaves. Macroscopic observations (left panel) and subsequent Evans blue stainings (right panel) are depicted 36 hai. Macroscopic symptoms of cell death were scored 36 hai: only infiltrations that resulted in confluent death of (nearly) the entire infiltrated region were scored and are presented (right panel) as a fraction of total infiltrations performed. Bars are 1 cm.

**Figure 3:**
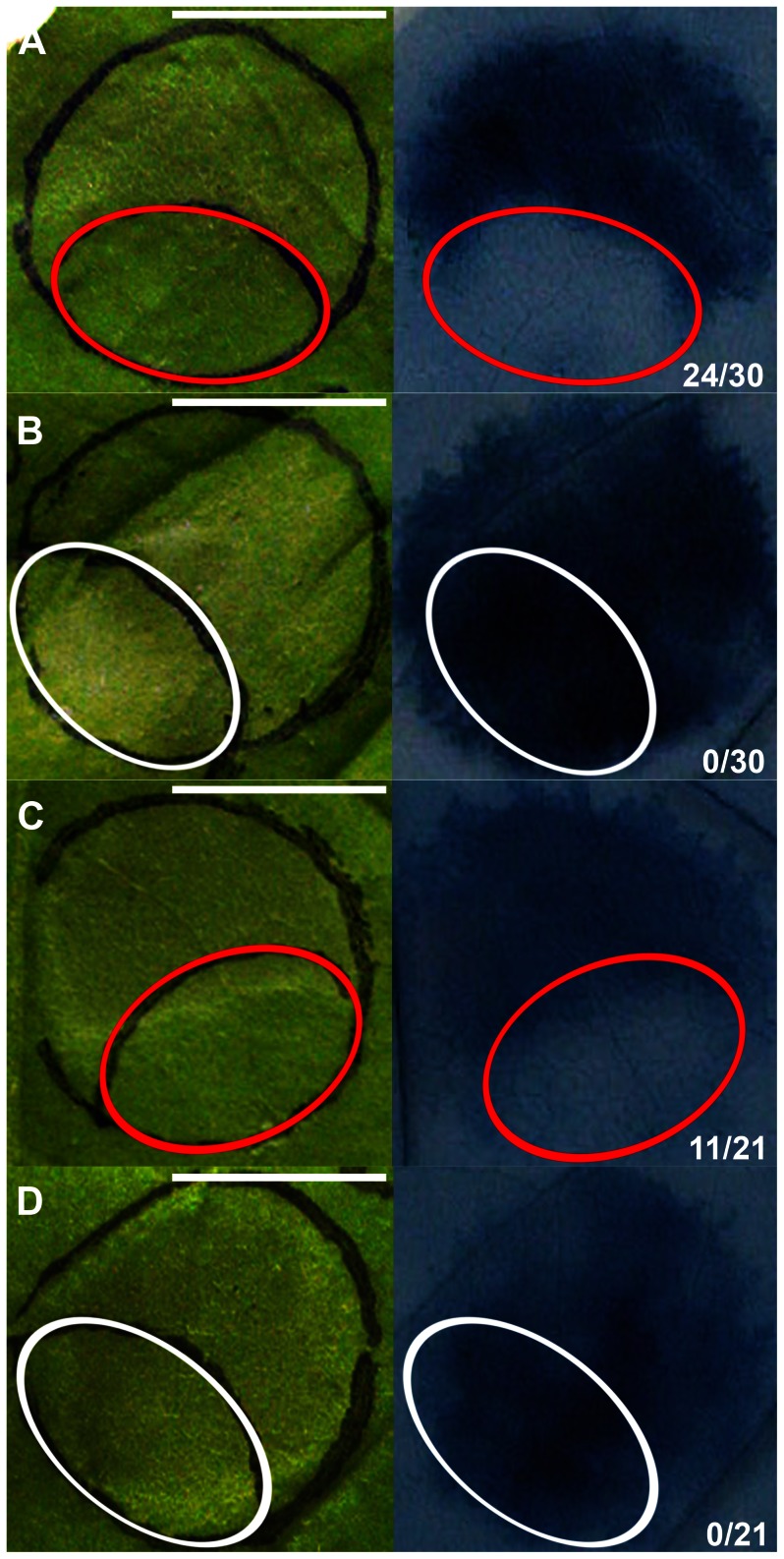
Lanthanum chloride delays the cell death development upon MtNFP and MtLYK3, or AtCERK1 (co-)production. *Agrobacterium* transformants carrying the following constructs were (co-)infiltrated at a final concentration: OD_600_ [*MtNFP-3xFLAG*] = 0.125 and OD_600_ [*MtLYK3-3xFLAG*] = 0.2 (A, B); OD_600_ [*AtCERK1-3xFLAG*] = 0.2 (C, D). Twelve hai parts of the infiltrated regions were syringe-infiltrated with 5 mM lanthanum chloride (circled in red) or water (circled in white). Macroscopic observations (left panel) and subsequent Evans blue stainings (right panel) are depicted 42 hai for leaf regions co-producing MtNFP and MtLYK3 fusions (A, B), and 33 hai for leaf regions producing AtCERK1 fusion (C, D). Cell death development was scored 42 hai (A, B) or 33 hai (C, D): only infiltrations that showed the lack of tissue collapse and no compromised membrane permeability in the lanthanum chloride- or water-treated region were scored and are presented (right panel) as a fraction of total infiltrations performed. Bars are 1 cm.

**Figure 4:**
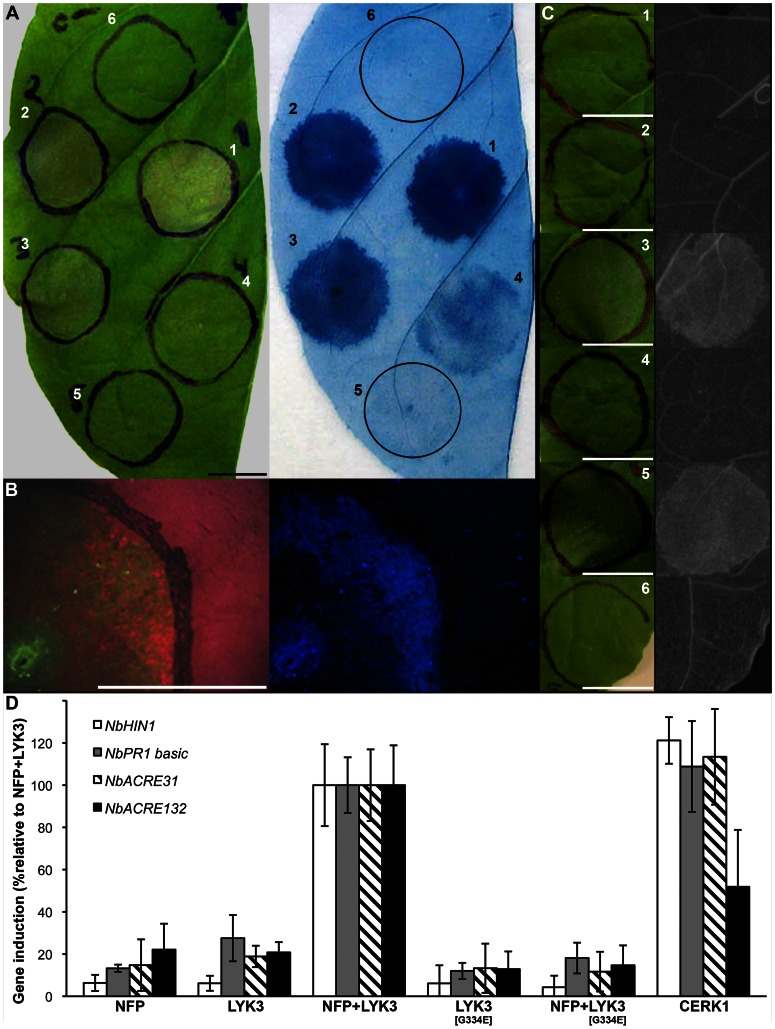
MtNFP and MtLYK3, or AtCERK1 (co-)production in *Nicotiana* leaves induces defence-like responses. A, Kinetics of cell death development in *Nicotiana*. *Agrobacterium* transformants carrying either *MtNFP-3xFLAG* or *MtLYK3-3xFLAG* construct were co-infiltrated into *Nicotiana* leaves at five different time points (1–5). Macroscopic observation (left panel) and subsequent Evans blue staining (right panel) are depicted 42 hai (region 1), 39 hai (region 2), 36 hai (region 3), 33 hai (region 4) and 30 hai (region 5). Mock infiltration (region 6) was done concomitantly with the infiltration of region 1. Bar is 1 cm. B, Changes in leaf autofluorescence upon MtNFP and MtLYK3 co-production. Leaf regions co-producing MtNFP-3xFLAG and MtLYK3-3xFLAG fusions were analyzed between 24 and 48 hai (here depicted 36 hai) using a stereoscope. Note the decrease in chlorophyll content, as indicated by the decrease of far-red autofluorescence of chlorophyll (left panel), and enhanced accumulation of blue light-excited autofluorescence (right panel) within the infiltrated region. Bar is 1 cm. C, Accumulation of phenolic compounds. The following fusions were (co-)produced in *Nicotiana* leaves: MtNFP-3xFLAG (1); MtLYK3-3xFLAG (2); MtNFP-3xFLAG+MtLYK3-3xFLAG (3); MtNFP-3xFLAG+MtLYK3[G334E]-3xFLAG (4); AtCERK1-3xFLAG (5); or AtCERK1[K349]-3xFLAG (6). Macroscopic observations (left panel) and subsequent UV-excited autofluorescence of ethanol/lactophenol-cleared (right panel) leaf regions are depicted 36 hai (except for 5–30 hai). Bars are 1 cm. D, Induction of *NbHIN1, NbPR1 basic*, *NbACRE31*, and *NbACRE132* expression in response to separate production or co-production of: MtNFP-3xFLAG (NFP), MtLYK3-3xFLAG (LYK3), MtLYK3[G334E]*-*3xFLAG (LYK3[G334E]), and AtCERK1-3xFLAG (CERK1). Leaf samples were collected 24 hai and induction of gene expression was analyzed using qRT-PCR. Histograms represent induction of *NbHIN1* (white columns), *NbPR1 basic* (grey columns), *NbACRE31* (hatched columns), and *NbACRE132* (black columns) normalized by one reference gene, *MtEF1 α*. Induction of each gene was normalized to that caused by mock infiltration, and then calculated as % induction relative to the induction observed upon co-production of MtNFP and MtLYK3 fusions. Bars represent standard deviation of the mean. At least two technical replicates from two biological replicates were analyzed.

**Figure 5:**
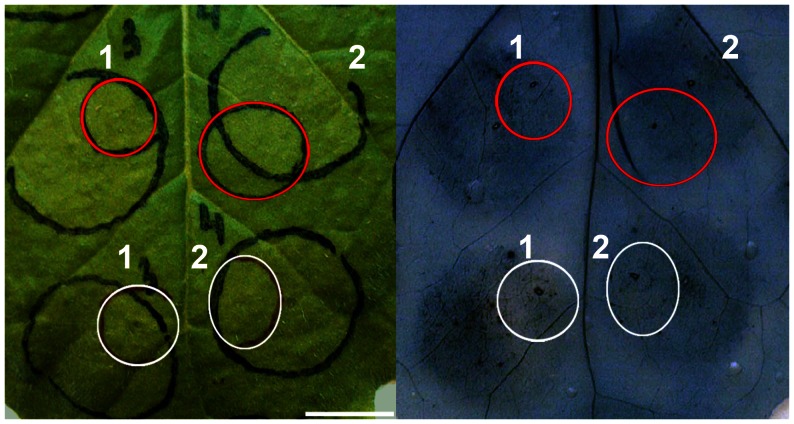
Cell death upon MtNFP and MtLYK3 co-production in *Nicotiana* leaves does not require *Sm*NF. *Agrobacterium* transformants carrying either *MtNFP-3xFLAG* or *MtLYK3-3xFLAG* construct were co-infiltrated into *Nicotiana* leaves at a final concentration: OD_600_ [*MtNFP*] = 0.25 and OD_600_ [*MtLYK3*] = 0.4 (1); OD_600_ [*MtNFP*] = 0.15 and OD_600_ [*MtLYK3*] = 0.25 (2). Twelve hai parts of the transformed regions were syringe-infiltrated with 10^−7 ^mM *Sm*NF (circled in red) or DMSO diluted to the same concentration (circled in white). Macroscopic observation (left panel) and Evans blue staining (right panel) are depicted 33 hai. Bar is 1 cm.

*Agrobacterium tumefaciens* GV3101::pMP90 and LBA4404 strains were transformed with the respective constructs via electroporation. The LBA4404 strain was used only in the experiments that compared the effect of different *Agrobacterium* strains on cell death (CD) induction upon MtNFP and MtLYK3 co-production. All results presented in Figures 1–5, Figure S1 and S3, and Tables 1–3 were obtained with *Agrobacterium* GV3101::pMP90 strain. *Agrobacterium*-mediated transformation of *Nicotiana* was performed essentially as described [42], except that *Agrobacterium* cultures were grown in LB medium supplemented with 25 µg/mL of rifampicin and 50 µg/mL of kanamycin. Resuspended cells were incubated at room temperature for at least 1 h before being infiltrated into fully expanded leaves of green house-grown plants using needleless syringes. *Agrobacterium* transformants carrying the respective construct were resuspended in the infiltration medium to desired OD_600_: all *MtNFP* and *MtNFP*[ΔInR]-*sYFP2* constructs - OD_600_ = 0.4; all *MtLYK3* and *AtCERK1* constructs - OD_600_ = 0.7; *MtDMI2-sYFP2* - OD_600_ = 1.0. Then, they were mixed 1∶1 with GV3101::pMP90 transformants carrying: pCambia1390 vector with an empty CaMV 35Sp::35S terminator cassette (for separate expression), a desired *MtNFP* construct or a desired *MtLYK3* construct before being infiltrated into *Nicotiana* leaves. All experiments included mock infiltration with GV3101::pMP90 transformants carrying pCambia1390 vector with an empty CaMV 35Sp::35S terminator cassette, and a positive control (co-expression of WT *MtNFP-FP* and WT *MtLYK3-FP* constructs). Cell death induction upon separate expression or co-expression of each (pair of) constructs was analyzed between 24 and 72 hai in at least three independent experiments, every time using three different plants. In case of no macroscopic symptoms, three leaves were stained with Evans blue to confirm the lack of CD.

**Table 1:** Cell death induction upon (co-)expression of various RLK-encoding genes in *Nicotiana* leaves.

Construct	Cell death induction		
	Separate expression	Co-expression with *MtNFP-FP #*	Co-expression with *MtLYK3-FP #*
*MtNFP*–*sYFP2*	0/12	Not applicable	20/22
*MtNFP* –*3xFLAG*	0/9	Not applicable	12/13*
*MtNFP*	0/9	Not applicable	8/9**
*MtLYK3*–*sYFP2*	0/12	20/22	Not applicable
*MtLYK3*–*3xFLAG*	0/9	12/13*	Not applicable
*MtLYK3*	0/9	8/9**	Not applicable
*MtDMI2*-*sYFP2*	0/12	0/12	0/12
*MtLRRII.1*-*YFP*_C_	0/9	0/9***	0/9***
*AtBRI1*- *YFP*_C_	0/7	0/9***	0/9***

# − unless stated differently: with *-3xFLAG* (*) untagged (**), or *−YFP*_N_ (***) tagged construct.

Indicated constructs were expressed alone or co-expressed with either *MtNFP* or *MtLYK3* in *Nicotiana* leaves, and the infiltrated regions were marked. Macroscopic symptoms of cell death were scored 48 hai: only infiltrations that resulted in confluent death of (nearly) the entire infiltrated region were scored and are presented as a fraction of total infiltrations performed.

**Table 2:** Cell death induction activity of MtNFP-sYFP2 truncated/mutated variants in *Nicotiana* leaves.

*MtNFP-sYFP2*construct	Subcellularlocalization*	Nodulationactivity*	Cell death induction	
			Co-expression with*MtLYK3-mCherry*	Separateexpression
WT	PM	+	28/30	0/12
S67F (*Mtnfp-2*)	ER	–	0/9	0/9
S67A	PM	+	6/9#	0/9
ΔInR	PM	–	0/20	0/9
G474E	partial PM	–	0/15	0/9

* -see [22], PM-plasma membrane, ER-endoplasmic reticulum.

The designated constructs were expressed alone or co-expressed with *MtLYK3-mCherry* construct in *Nicotiana* leaves. Macroscopic symptoms of cell death were scored 48 hai: only infiltrations that resulted in confluent death of (nearly) the entire infiltrated region were scored and are presented as a fraction of total infiltrations performed. # - In the 3 remaining leaf regions, co-expression of *MtNFP*[S67A]-*sYFP2* and *MtLYK3-mCherry* constructs resulted in increased staining with Evans blue in the entire infiltrated region.

**Table 3:** Cell death induction activity of MtLYK3-sYFP2 mutated variants in *Nicotiana* leaves.

*MtLYK3-sYFP2* construct	Auto-phosphorylation activity*	Nodulation activity**	Cell death induction	
			Co-expression with *MtNFP-mCherry*	Separate expression
WT	+	+	28/30	0/12
P87S (Mtlyk3-3)	Not applicable	-	15/15	0/9
T319A	–	–	0/11	0/9
G334E (Mtlyk3-1)	–	–	0/20	0/9
K349A	–	–	0/16	0/9
E362A	–	–	0/15	0/9
D441A	–	–	0/16	0/9
T475A	–	–	0/13	0/9
T480A	–	−(0/24)	0/18	0/9
T285A/S286A/T300A	+	Reduced with T300A	15/16	0/9
T433A	+	Reduced	Reduced 7/20	0/10
K464A	Reduced	Reduced (7/18)	0/12#	0/9
S471A	+	Reduced	9/11	0/9
T472A	+	Reduced	Reduced 5/11	0/9
T512A	Reduced	–	Reduced 12/20	0/9

* -see [20], except for the T480A (Fig. S2), **-see [20], except for the P87S [12], K464A and T480A (this study; number of plants nodulated/number of plants tested).

The designated constructs were expressed alone or co-expressed with *MtNFP-mCherry* construct in *Nicotiana* leaves. Macroscopic symptoms of cell death were scored 48 hai: only infiltrations that resulted in confluent death of (nearly) the entire infiltrated region were scored and are presented as a fraction of total infiltrations performed. # - despite the lack of pronounced macroscopic symptoms, the co-expression of *MtLYK3*[K464A]-*sYFP2* and *MtNFP-mCherry* constructs resulted in increased staining with Evans blue in the entire infiltrated region.

To confirm efficient accumulation of MtLRRII.1-YFP_C_ and AtBRI1-YFP_C_ fusions in *Nicotiana*, *Agrobacterium* transformants carrying the respective constructs were co-infiltrated at high optical densities (final OD_600_ = 0.5) with *Agrobacterium* transformants carrying either *MtNFP-YFP*_N_ or *MtLYK3-YFP*_N_ constructs (final OD_600_ = 0.5). The observed complementation of YFP fluorescence reported on efficient accumulation, and even unspecific oligomerization of the respective encoded fusions.

### Stereoscopic Analysis

Blue light-excitable autofluorescence and far-red chlorophyll autofluorescence in intact *Nicotiana* leaves were imaged using 430/40 excitation and 485/50 emission BP filters, or 480/40 BP excitation and 510 LP emission filters, respectively. Images were captured using CMOS USB DCC1645C camera (THORLabs, Newton NJ, USA) implemented on a Leica MZ FLIII stereoscope. Evans blue staining was performed as described [42]. Leaves were cleared by boiling in acidic lactophenol/ethanol solution (10 g phenol in 10 ml lactic acid, mixed 2∶1 with 96% ethanol) until the complete removal of chlorophyll (approximately 3 min per leaf). Ethanol-inextractable autofluorescence was excited with 312 nm wavelength. Images were captured using a Cool Snap CF camera (Photometrix, Tucson AZ, USA).

### qRT-PCR Analysis

RNA extraction and qRT-PCR were performed as described [17] except that cDNA was prepared from 500 ng of total RNA (see Table S1 for primer sequences). Two technical replicates from two biological replicates were analyzed and results were collated.

### *Medicago* Transformations

Complementation of *Mtlyk3-1* mutant seedlings was performed as described [20] using *MtLYK3-3xFLAG*, *MtLYK3*[K464A]-*3xFLAG*, and *MtLYK3*[T480A]-*sYFP2* constructs driven by the CaMV 35S promoter. Results were scored as:+(>75% of plants nodulated), reduced (<50% of plants nodulated) or - (0 plants nodulated).

## Results

### Co-production of MtNFP and MtLYK3 in *Nicotiana* Leaves Induces Cell Death

*MtNFP* and *MtLYK3* cDNA sequences were fused at their 3′ ends to the sequence encoding a fluorescent protein (FP); either a super yellow fluorescent protein 2 (sYFP2) or mCherry [43]–[44], and were expressed from a CaMV 35S promoter in *Nicotiana* leaves, where they were delivered by *Agrobacterium-*mediated transformation. These and similar *MtNFP* and *MtLYK3* constructs were shown to complement *Mtnfp* and *Mtlyk3* mutants, respectively [17]–[18], [22], and are therefore suitable for studying the encoded LysM-RLKs. Confocal laser-scanning microscopy analysis demonstrated co-localization of both MtNFP-sYFP2 and MtLYK3-sYFP2 fusions with a plasma membrane (PM) marker (the hyper-variable region [HVR] of maize [*Zea mays*] ROP7 fused to the C-terminus of mCherry; [20], [22]), hence indicating PM localization of MtNFP and MtLYK3 fusions in *Nicotiana* leaf epidermal cells. Surprisingly, co-infiltration of *Agrobacterium* transformants carrying *MtNFP-FP* or *MtLYK3-FP* constructs, leading to the co-production of the encoded fusions, resulted in collapse and subsequent desiccation of the infiltrated region within 48 hours after infiltration (hai) (Fig. 1A), regardless of the *Agrobacterium* strain used (i.e. GV3101::pMP90 or LBA4404; unpublished data). This cell death (CD) response was not dependent on the tag attached to either protein, since an identical response was observed upon co-production of FP-tagged, 3xFLAG-tagged or untagged MtNFP and MtLYK3 (Fig. 1A, Table 1). Importantly, production of the separate MtNFP or MtLYK3 (fusions) did not induce CD as confirmed with an exclusion dye (Evans blue) staining (Fig. 1A), which reflects compromised membrane permeability attributed with cell death.

To investigate whether a similar CD response could be triggered by heterologous production of other plant RLKs, we analysed the *Nicotiana* response to expression of *Medicago Doesn’t Make Infection 2* (*DMI2*; [45]), *MtLRRII.1* [41]), and *Arabidopsis Brassinosteroid Insensitive 1* (*BRI1*; [46]), all driven by the CaMV 35S promoter. Notably, none of these RLKs, alone or in combination with either MtNFP or MtLYK3 fusions, induced CD (Fig. 1B, Table 1), despite being efficiently produced in *Nicotiana* leaves, as confirmed with fluorescence microscopy (see Materials & Methods). Thus, the *Nicotiana* CD response was not a general response to a heterologous production of RLKs but rather a specific response to MtNFP and MtLYK3 co-production.

### Production of AtCERK1 also Induces Cell Death in *Nicotiana* Leaves

A rapid tissue collapse at the site of pathogen attack, termed the hypersensitive response (HR), is frequently observed in incompatible plant-pathogen interactions where it is thought to contribute to pathogen restriction and to generate a signal that activates systemic plant defence mechanisms [47]–[48]. The apparent phenotypic similarity of the CD response to MtNFP and MtLYK3 co-production with the HR elicited by various pathogen-derived components (MAMPs and so-called effectors) [49]–[50], prompted us to investigate whether co-production of the symbiotic LysM-RLKs might activate defence signalling similar to that mediated by LysM-RLKs functioning in innate immunity. AtCERK1 mediates signalling upon the perception of COs or PGN, although, to our knowledge, CD induction in response to these MAMPs has not been reported so far in any plant species. We therefore investigated the *Nicotiana* response to heterologous production of wild-type (WT) AtCERK1 or its kinase-inactive variant carrying a substitution in the catalytic lysine (Lys 349 in a kinase subdomain II). Both *AtCERK1* and *AtCERK1*[K349E] constructs were generated as fusions to the 5′ end of the *sYFP2* sequence, and their expression in *Nicotiana* leaves was driven by the CaMV 35S promoter.

Notably, heterologous production of AtCERK1-sYFP2 fusion resulted in tissue collapse and desiccation of (nearly) the entire infiltrated region in 20 out of 22 infiltrations 36 hai (Fig. 2A). This CD induction abolished our attempts of precisely characterizing the subcellular localization of AtCERK1 fusion in *Nicotiana* leaf epidermal cells, although we could detect sYFP2 fluorescence at the cell boundary (unpublished data). On the contrary, we observed clear co-localization of AtCERK1[K349E]-sYFP2 fusion with the PM marker using confocal laser-scanning microscopy analysis (Fig. S1). The PM localization of AtCERK1[K349E] fusion in *Nicotiana* leaf epidermal cells is in agreement with the reported subcellular localization of AtCERK1 fluorescent fusion in onion (*Allium cepa*) epidermal cells [31]. Importantly, production of the kinase-inactive variant of AtCERK1 did not result in CD induction, as confirmed with Evans blue staining (Fig. 2B), indicating that biological activity of AtCERK1 in *Nicotiana* leaves was dependent on its kinase activity.

### Cell Death Induction Upon MtNFP and MtLYK3 Co-production, and AtCERK1 Production in *Nicotiana* Leaves Requires an Influx of Extracellular Ca^2+^

An influx of extracellular Ca^2+^ causes an increase in the cytosolic [Ca^2+^] that is required for MAMP (including COs)-induced activation of a MAPK cascade, ROS production, and gene expression. Thus, Ca^2+^ influx is postulated to occur very early in the plant defence signalling pathway [51]–[52], possibly immediately upon the activation of the PM-localised MAMP receptors [53] We wanted to know whether an influx of extracellular Ca^2+^ was similarly involved in CD induction upon MtNFP and MtLYK3 co-production or separate production of AtCERK1. To this end, MtNFP-3xFLAG and MtLYK3-3xFLAG fusions or AtCERK1-3xFLAG fusion were (co-)produced in adjacent regions in *Nicotiana* leaves. Twelve hours later, parts of the infiltrated leaf regions were syringe-infiltrated with 5 mM lanthanum chloride (an established inhibitor of the PM-localized calcium channels) or water, and the CD development was monitored between 24 and 72 hours after the first infiltration (with *Agrobacterium*). Notably, in 24 out of 30 leaf regions co-producing MtNFP and MtLYK3 fusions, compromised membrane permeability and tissue collapse were first (i.e. between 36 and 42 hai) localized only (or mostly) outside the lanthanum chloride-treated regions (Fig. 3A). Later on (i.e. 60 hai), 26 out of 30 parts of leaf regions treated with lanthanum chloride showed confluent death of the entire infiltrated region (unpublished data). Similar delay of the CD development was observed 33 hai in 11 out of 21 leaf regions producing AtCERK1 fusion and treated with lanthanum chloride (Fig. 3C). On the contrary, control treatment with water did not affect the development of confluent CD upon (co-)production of MtNFP and MtLYK3 fusions or AtCERK1 fusion (Fig. 3B, D).

### Cell Death Upon MtNFP and MtLYK3 Co-production, and AtCERK1 Production in *Nicotiana* Leaves is Associated with an Induction of Defence-like Responses

Subsequently, we investigated whether co-production of MtNFP and MtLYK3 or production of AtCERK1 in *Nicotiana* leaves was associated with an accumulation of phenolic compounds and/or induction of defence-related gene expression, two established hallmarks of plant defence response, including that induced by COs and/or PGN [54]–[56]. We started by analysing the kinetics of CD development. To this end, *Agrobacterium* transformants carrying *MtNFP-3xFLAG*, *MtLYK3-3xFLAG* or *AtCERK1-3xFLAG* construct were (co-) infiltrated at different time-points in adjacent circles in *Nicotiana* leaves, and CD development was monitored between 24 and 48 hai. In case of co-production of MtNFP and MtLYK3 fusions, macroscopic symptoms of CD were first observed around 36 hai (Fig. 4A) as a type of flaccidity and the appearance of small patches of collapsed tissue (these were more pronounced on the abaxial side of the leaf). Forty-eight hai, 30 out of 31 infiltrations showed pronounced tissue desiccation of the entire infiltrated region (Fig. 1A). Compromised membrane permeability preceded tissue collapse and often occurred over the entire infiltrated region approximately 33 hai (Fig. 4A). Compromised membrane permeability of leaf regions producing AtCERK1 fusion was observed already approximately 27–30 hai, and pronounced macroscopic symptoms of CD developed 36 hai (Fig. 2A, Fig. 3 C, D).

In addition, co-production of MtNFP-3xFLAG and MtLYK3-3xFLAG fusions resulted in accumulation of blue light-excitable autofluorescence (Fig. 4B) approximately 36 hai. This was not observed upon separate production of either fusion, or upon co-production of MtNFP-3xFLAG and kinase-inactive MtLYK3[G334E]-3xFLAG fusions (unpublished data). Accumulation of ethanol/lactophenol-inextractable and UV-excitable autofluorescence, indicative of phenolic compounds, was detected approximately 36 hai and 30 hai in leaf regions (co-)producing MtNFP-3xFLAG and MtLYK3-3xFLAG fusions or AtCERK1-3xFLAG fusion, respectively (Fig. 4C). Mock infiltration, separate production of MtNFP-3xFLAG or MtLYK3-3xFLAG fusion, production of kinase-inactive AtCERK1[K349E]-3xFLAG fusion or co-production of MtNFP-3xFLAG and kinase-inactive MtLYK3[G334E]-3xFLAG fusions did not result in the accumulation of similar autofluorescence (Fig. 4C).

Subsequently, we investigated induction of defence-related genes expression in *Nicotiana* leaves in response to: separate production and co-production of MtNFP-3xFLAG, MtLYK3-3xFLAG, MtLYK3[G334E]-3xFLAG, and AtCERK1-3xFLAG fusion(s). Induction of: *NbHIN1*– a postulated marker gene for HR [50]; two *PR1* genes, i.e. *NbPR1a acidic* and *NbPR1 basic* [57]; and *NbACRE31*, *NbACRE132*, and *NbCYP71D20*–postulated marker genes for MAMP-triggered immunity [49] was analyzed 24 hai using quantitative reverse-transcriptase polymerase chain reaction (qRT-PCR). Co-production of MtNFP and MtLYK3 fusions, and separate production of AtCERK1 fusion resulted in an induction of *NbHIN1*, *NbPR1 basic*, *NbACRE31*, and *NbACRE132* gene expression that was significantly higher than that following co-production of MtNFP and MtLYK3[G334E] fusions or separate production of MtNFP, MtLYK3, and MtLYK3[G334E] fusions (Fig. 4D). The *NbPR1a acidic* and *NbCYP71D20* genes did not display significant induction upon (co-) production of any of the protein(s) tested (unpublished data).

Taken together, the indication of a localized accumulation of phenolic compounds and induction of defence-related gene expression suggested that the *Nicotiana* response to MtNFP and MtLYK3 co-production triggered responses that were qualitatively similar to the responses to heterologous production of AtCERK1. In addition, the fact that in both cases the impairment of Ca^2+^ influx delayed the CD development suggested that the apparent functional interaction of two symbiotic LysM-RLKs in *Nicotiana* leaves mimics the action of the MAMP receptor, AtCERK1, and triggers defence-like responses.

### Cell Death Induced in *Nicotiana* Leaves Upon MtNFP and MtLYK3 Co-production is a NF-independent Response

Perception of NFs results in triggering host symbiotic programme mediated by MtNFP and/or MtLYK3 [2]. In contrast, co-production of these LysM-RLKs in *Nicotiana* leaves apparently triggered some signalling cascade in the absence of NFs. Therefore, we investigated the effect of NF produced by *Sinorhizobium meliloti*, a microsymbiont of *Medicago*, on this CD response. To this end, *Agrobacterium* transformants carrying either *MtNFP-sYFP2* or *MtLYK3-mCherry* construct were co-infiltrated into *Nicotiana* leaves at varying concentrations (as measured with OD_600_). Then, purified *Sm*NF at 10^−7 ^M (in diluted DMSO) or diluted DMSO alone was applied between 9 and 24 hai to parts of the leaf regions co-producing MtNFP and MtLYK3 fusions, and CD development was monitored between 24 and 72 hours after the first infiltration (with *Agrobacterium*) using Evans blue staining. For all bacterial concentrations and time-points of *Sm*NF/DMSO application tested, compromised membrane permeability in leaf regions co-producing MtNFP and MtLYK3 fusions was observed at similar time irrespective of the *Sm*NF or DMSO treatment (Fig. 5), indicating similar kinetics of CD development. Therefore, we did not obtain evidence for any stimulatory or inhibitory effect of the *Sm*NF on the CD development upon MtNFP and MtLYK3 co-production.

### The Intracellular Region of MtNFP and Kinase Activity of MtLYK3 are Required for Cell Death Induction in *Nicotiana* Leaves

The independence of CD induction upon MtNFP and MtLYK3 co-production from the *Sm*NF perception prompted us to compare structural requirements of CD induction and nodulation with regard to these LysM-RLKs. In case of MtNFP, a recent structure-function study in *Medicago* [22] showed that loss-of-function mutations located in the ExR could be attributed to retention of the mutated protein in the endoplasmic reticulum (ER), whereas most substitutions located in the InR were found not to have an effect on the MtNFP function in nodulation. Therefore, we decided to limit our analysis of MtNFP to three point-mutated variants carrying: Ser 67 Phe (encoded by the *Mtnfp-2* allele), Ser 67 Ala, and Gly 474 Glu substitution; and a truncated variant with almost the entire InR deleted, termed MtNFP[ΔInR] (amino acids: 1–283) (see Table 2). Based on structure-function studies on MtLYK3 and LjNFR1, respectively in *Medicago* and in *Lotus* ([12], [20]–[21], Table 3 in this study), we decided to test the effect of 16 point mutations (listed in Table 3) on MtLYK3 ability to induce CD in *Nicotiana* leaves in the presence of MtNFP. These included: a Pro 87 Ser (encoded by the *Mtlyk3-3* allele) and a Gly 334 Glu (encoded by the *Mtlyk3-1* allele) mutations, and Ala substitutions of Thr 285, Ser 286, Thr 300, Thr 319, Lys 349, Glu 362, Thr 433, Asp 441, Lys 464, Ser 471, Thr 472, Thr 475, Thr 480, and Thr 512. With the exception of the P87S substitution located in the first LysM domain of MtLYK3, all the above mutations are located in the MtLYK3 InR but differ in their effect on MtLYK3 autophosphorylation activity *in vitro* ([20] and Fig. S2; see Table 3). All truncated/mutated variants were prepared as fusions to the N-terminus of sYFP2, and their production and correct PM localization in *Nicotiana* leaf epidermal cells was confirmed, except for two MtNFP variants: MtNFP[S67F]-sYFP2 fusion was retained in the ER, and MtNFP[G474E]-sYFP2 fusion showed a partial PM localization, ([20], [22] and Fig. S1). Additionally, we ruled out a possibility that the presence of WT MtNFP-FP or WT MtLYK3-FP fusion might affect stability/localization of the truncated/mutated fusions by confirming their efficient production and PM localization in *Nicotiana* leaf epidermal cells also upon co-production with MtLYK3 or MtNFP fusions (unpublished data).

Subsequently, we analyzed the ability of truncated/mutated MtNFP and MtLYK3 variants to induce CD upon either their separate production or co-production with WT MtLYK3-mCherry or WT MtNFP-mCherry fusion, respectively. In order to compare the CD induction ability of truncated/mutated variants with WT proteins, concomitant co-infiltration with *Agrobacterium* transformants carrying either WT *MtNFP-FP* or WT *MtLYK3-FP* construct was done on every leaf. Development of CD was monitored between 36 and 72 hai, and in case of the absence of or weakly pronounced macroscopic symptoms, the occurrence of CD was further scrutinized with Evans blue staining. None of the truncated/mutated variants was able to induce CD in *Nicotiana* leaves on its own (Table 2, 3). Co-production of MtNFP[S67A]-sYFP2 and MtLYK3 fusions resulted in a confluent death of (nearly) the entire infiltrated region in 6 out of 9 infiltrations, and compromised membrane permeability that could be observed in the entire infiltrated region (Table 2). In contrast, co-production of MtLYK3 fusion with MtNFP[S67F]-sYFP2, MtNFP[G474E]-sYFP2, or MtNFP[ΔInR]-sYFP2 fusion did not induce CD in *Nicotiana* leaves (Table 2). In case of MtLYK3 mutated variants, co-production of MtLYK3[P87S]–sYFP2 and MtNFP fusions induced confluent CD in all infiltrated regions (Table 3). In contrast, co-production of MtNFP fusion with all seven MtLYK3-sYFP2 mutated variants affected for their autophosphorylation activity *in vitro* did not induce CD in *Nicotiana* leaves (Table 3). In case of mutations that do not affect autophosphorylation activity of MtLYK3 kinase, we found that MtLYK3[T285A S286A T300A]-sYFP2 and MtLYK3[S471A]-sYFP2 fusions were as active as WT MtLYK3-sYFP2 fusion for CD induction upon their co-production with MtNFP fusion, whereas MtLYK3[K464A]-sYFP2 fusion induced compromised membrane permeability (but no macroscopic symptoms of cell death) upon co-production with MtNFP fusion (Fig. S3, Table 3). Co-production of MtNFP fusion with MtLYK3[T433A]-sYFP2, MtLYK3[T472A]-sYFP2 or MtLYK3[T512A]-sYFP2 fusion resulted in a confluent death of (nearly) the entire infiltrated region in, respectively, 7 out of 20, 5 out of 11, and 12 out of 20 infiltrations, whereas the remaining leaf regions displayed only (a) small patch(es) of dead tissue (Fig. S3, Table 3).

Taken together, most of the structural requirements regarding the MtNFP and MtLYK3 InR, and the autophosphorylation activity of the MtLYK3 KD, appeared to be identical for biological activity of these LysM-RLKs in both *Medicago* and *Nicotiana*. More specifically, we found out that both nodulation and CD induction displayed the same requirements for 11 out of 15 residues located in the MtLYK3 InR. On the contrary, a single mutation in the MtLYK3 ExR tested (that does not affect the PM localization of the fusion) was found to be crucial for MtLYK3 function in nodulation but not in CD induction. In case of MtNFP, the substitution of Ser 67 similarly abolished (S67F) or did not have an effect (S67A) on MtNFP function in nodulation and CD induction, which seemed to correlate with, respectively, the absence or presence of MtNFP fusion at the PM.

## Discussion

### Co-production of MtNFP and MtLYK3 in *Nicotiana* Induces Defence-like Responses that Resemble *Nicotiana* Responses to AtCERK1 Production

Efficient production of both MtNFP-FP and MtLYK3-FP fusions *in Nicotiana* leaves facilitated characterization of their subcellular localization [20], [22] and oligomerization status *in vivo* (manuscript in preparation), and led to the surprising observation of a CD induction (Fig. 1A). This response phenotypically and kinetically (Fig. 4A) resembled the HR elicited in *Nicotiana* spp. by pathogen-derived components [49], or CD induced by (co-) production of certain defence-related proteins, e.g. Pto [58]. Notably, a similar CD response was observed in *Nicotiana* leaves upon production of AtCERK1 (Fig. 2A), a MAMP receptor. Although CD induction was not demonstrated in any plant species in response to COs or PGN [54]–[56], it has been observed upon deregulation of various stress/defence-related signalling components [59]–[60], including a mitogen-activated protein kinase kinase (OsMKK4) implicated in COs-induced signalling in rice [61]. In addition, expression of *AtCERK1* from the CaMV 35S promoter (used also in our studies) in *Arabidopsis* was shown to result in a ligand-independent dimerization of AtCERK1 [38]. We hypothesize that overproduction of this LysM-RLK in *Nicotiana* analogously leads to its dimerization and enhanced (deregulated) kinase activity, which in turn is required and sufficient for triggering CD; a response not observed upon ligand-induced activation of AtCERK1 in *Arabidopsis*.

Cell death induction upon MtNFP and MtLYK3 co-production in *Nicotiana* is in agreement with the *Nicotiana* [21] and *Arabidopsis* [19] response to LjNFR1 and LjNFR5 co-production. However, in these studies the associated induction of putative defence-related responses has not been investigated. We here showed that both MtNFP and MtLYK3 co-production and AtCERK1 production in *Nicotiana* leaves triggered local accumulation of phenolic compounds, and a similar induction of expression of 4 out of 6 tested defence-related genes (Fig. 4C, D). We speculate that the two other genes might display different kinetics of induced expression (here analyzed only 24 hai) or might undergo suppression by *Agrobacterium* [62]. Importantly, COs- and/or PGN-induced expression of *PR1*, *ACRE31*, *ACRE132* and a member of a *HIN1* gene family was reported previously [32], [52], [54], linking these genes to MAMP-induced gene regulation mediated by AtCERK1 in *Arabidopsis* and/or *NbCERK1* in *Nicotiana*. In addition, we found that the lanthanum chloride-induced impairment of a Ca^2+^ influx similarly delayed the CD development upon (co-)production of the LysM-RLKs in our study (Fig. 3A, C). Therefore, we speculate that the signalling triggered upon MtNFP and MtLYK3 co-production in *Nicotiana* mimics AtCERK1-mediated signalling and thereby results in an induction of defence-like responses.

### Similarities and Differences between Symbiotic and Defence Signalling Mediated by LysM-RLKs

The similarity between *Nicotiana* response to MtNFP and MtLYK3 co-production and AtCERK1 production suggests a possible overlap in signalling mediated by these LysM-RLKs. Several NF-induced processes, such as: a transient increase of reactive oxygen species (ROS) production; activation of phospholipase C (PLC) and PLD; and prolonged oscillations of (peri)nuclear [Ca^2+^] are implicated in switching on the symbiotic programme [63]–[69], whereas a Ca^2+^ influx is postulated to act as a signal for infection thread formation [70]. Interestingly, (CERK1-mediated) COs- and/or PGN-induced responses also involve a Ca^2+^ influx, an elevated ROS production, and PLC activation [33], [35], [37]–[38], [52]–[53], [71]–[73]. We speculate that these similar processes might be activated/regulated by related molecular components, hence allowing two *Medicago* LysM-RLKs to activate signalling components present in *Nicotiana* leaf. Remarkably, Nakagawa and associates [74] demonstrated that swapping of the AtCERK1 ExR and a certain part of the AtCERK1 InR for the corresponding regions from LjNFR1 conferred on AtCERK1 a competence, albeit inefficient, for symbiotic signalling during *Lotus*-*Mesorhizobium loti* interaction. Conversely, our results demonstrate that MtNFP and MtLYK3, when co-produced in *Nicotiana*, are capable of signalling in a similar manner to AtCERK1. We hypothesize that due to the absence of symbiosis-specific “decoders” or “modulators” in *Nicotiana*, MtNFP- and MtLYK3-mediated signalling might be differently interpreted in this species, resulting in the induction of defence-like responses.

Importantly, NF-induced host responses are partially contradictory. On one hand, NFs are postulated to suppress the production in legume roots of salicylic acid and ROS, two potent signals implicated in defence signalling, upon rhizobia perception [63], [75]. This differential induction of the symbiotic competence or defence could be regulated quantitatively via NPR1 (*Non-expressor of Pathogenesis-related genes 1*)-mediated signalling [76]. On the other hand, even perception of compatible NFs leads to the induction of (some) defence-related gene expression and phosphorylation of defence-related proteins in the initial stage of symbiosis [7], [11], [68], [74]. An even more striking example of a NF-induced defence-like response comes from *Sesbania rostrata*. NF produced by its microsymbiont, *Azorhizobium caulinodans*, triggers the production of hydrogen peroxide and ethylene that eventually lead to local CD required for the formation of cortical infection pockets [77]. Therefore, our results agree with the hypothesized stimulation of (initial) host’s defence responses via LysM-RLKs that mediate NF-induced signalling.

### Possible Co-functioning of MtNFP and MtLYK3 in *Nicotiana* and *Medicago*

To explain a possible signalling mechanism employed by the kinase-inactive MtNFP, it has been proposed to form a receptor complex with MtLYK3 and another LYK protein during, respectively, the infection thread growth and pre-infection stage of symbiosis [12], [15]. In contrast, it is not known whether MtNFP functions alone during arbuscular mycorrhiza symbiosis [24]–[26] or resistance towards fungal and oomycete pathogens [27] or requires a similar co-functioning with (an)other RLK(s). We here demonstrated that MtNFP required MtLYK3 to induce CD in *Nicotiana*, and that neither of these LysM-RLKs could be substituted by an unrelated RLK (Fig. 1B, Table 1). We propose that this *Nicotiana* response reflects a functional interaction between these LysM-RLKs. A rather limited heteromerization of MtNFP and a kinase-inactive MtLYK3 variant observed in the PM of *Nicotiana* leaf epidermal cells (manuscript in preparation) does not exclude either hypothesized mechanism: a direct, phosphorylation-dependent physical interaction between MtNFP and WT MtLYK3; or indirect (functional) interaction between MtNFP and MtLYK3 that requires independent activation of different molecular components by either LysM-RLK, and a later convergence of such putative signalling pathways. Interestingly, MtLYK3, but not the homologous [78] AtCERK1, required the presence of MtNFP for CD induction in *Nicotiana* (Fig. 2A), indicating the specific requirement for MtNFP to potentiate the MtLYK3-mediated signalling. This observation agrees with the hypothesized specialization of the LysM-RLKs mediating NF-induced signalling during the co-evolution of legumes with rhizobia [28].

### Cell Death Induction in *Nicotiana* and Nodulation in *Medicago* Share Certain Structural Requirements Regarding MtNFP and MtLYK3

Curiously, CD induction in *Nicotiana* was independent from the NF perception (Fig. 5), and the presence of Pro 87 in the MtLYK3 ExR (Table 3), in contrast to MtNFP and MtLYK3 function in nodulation [12], [15]. Further mapping of crucial amino acid residues, and detailed characterization of their exact role in signalling would be required to clarify in the future whether or not nodulation and CD induction indeed hold different structural requirements with regard to the ExRs of these LysM-RLKs. On the contrary, the biological activity of MtNFP in *Nicotiana* was dependent on its PM localization, the presence of its InR, and the Gly 474 (Table 2), thus mimicking the structural requirements of RL symbiosis regarding MtNFP [22], and proteins encoded by *MtNFP* orthologs in *Lotus* [79] and pea (*Pisum sativum*) [5]. The overlap between structural requirements of nodulation and CD induction was even more pronounced with respect to the MtLYK3 InR. Out of 16 residues whose role in nodulation was identified, 9 residues (Thr 319, Gly 334, Lys 349, Glu 362, Thr 433, Asp 441, Thr 472, Thr 475, and Thr 480) were found to be equally important, and 2 residues (Thr 285, Ser 286) were equally dispensable for MtLYK3 biological activity both in *Medicago* and in *Nicotiana* (Table 3). In addition, the K464A and T512A substitutions had a negative effect of MtLYK3 biological activity in both *Nicotiana* and *Medicago*, although this effect was more (K464A) or less (T512A) severely pronounced during CD induction assays than during nodulation (Table 3). Various mutations abolishing MtLYK3 autophosphorylation activity ([20] and Fig. S2) similarly abolished its biological activity in *Medicago* [20] and in *Nicotiana* (Table 3), supporting the hypothesis that autophosphorylation of MtLYK3 is crucial for its signalling function. Importantly, as the role of Thr 480 in nodulation has not been described so far, our results revealed its importance for MtLYK3 function *in vivo*. Notably, the shared structural requirements of nodulation and CD induction were also confirmed with regard to several (putative) phosphorylation sites that do not abolish MtLYK3 autophosphorylation activity *in vitro* (Table 3). Phosphorylation within the InR of a RLK is often required for activation and regulation of its catalytic activity, and for generation of docking sites for (downstream) signalling components [80]–[82]. The shared importance of three (Thr 433, Thr 472, and Thr 512) out of five such phosphorylation sites for MtLYK3 biological activity in both plant species suggests that some of these phosphorylation-dependent functions required for MtLYK3-mediated signalling are conserved in *Nicotiana* leaf.

Demonstrated significant overlap between structural requirements of nodulation and CD induction regarding the MtNFP and MtLYK3 InRs supports our notion of the relevance of the *Nicotiana* system for studies on these, and potentially other (symbiotic) LysM-RLKs. This system presents certain practical advantages over the legume root system, in terms of rapidity and ease of expression of multiple constructs. In view of hypothesized similarities between NF (i.e. lipo-chitooligosaccharide)-induced and COs-induced signalling, analyzing known molecular components/processes involved in the CERK1-mediated signalling [35]–[38], [83] might provide information on the yet-unidentified players implicated in the perception and/or transduction of the NF signal. This would be especially important as still very little is known about the identity of interactors of these symbiotic LysM-RLKs [17]–[18], [21], [84]. Possible candidate signalling molecule(s) functioning in co-operation with, or downstream from the LysM-RLKs, and identified in this heterologous system should then be evaluated in legume root in order to confirm their involvement in symbiosis.

## Supporting Information

Figure S1 **Subcellular localization of various protein fusions in** ***Nicotiana*** **leaf epidermal cells.** The plasma membrane marker, mCherry-HVR, was co-produced with the designated fusions in *Nicotiana* leaf epidermal cells, and the fluorescence (viewed from abaxial side) was imaged 24 hai using confocal laser scanning microscopy. From left to right: green fluorescence of sYFP2; orange fluorescence of mCherry; superimposition of green, orange, and far-red (chlorophyll) fluorescence with the differential interference contrast (DIC) image. Bars are 20 µm. Note 1: in case of subcellular localization of MtNFP[G474E]-sYFP2 fusion, strong fluorescent puncta (indicated with an arrowhead) at the cell boundary of many cells (sometimes in association with nuclei), and pronounced ER localization (indicated with an arrow) of the fusion were still visible at 48 hai. Nevertheless, some cells showed a more uniform pattern of fluorescence at the cell boundary, and this observation, together with a partial insensitivity of this mutated variant to the PNGaseF treatment [22], indicated that at least some MtNFP[G474E] fusion had reached the PM. Note 2: as all kinase-inactive MtLYK3 variants were produced and correctly localized to the plasma membrane in *Nicotiana* leaf epidermal cells, their lack of biological activity can be attributed to the general abolishment of kinase activity rather than to an individual effect of a particular mutation.
(TIF)

Figure S2 **Effect of the Thr 480 Ala substitution on MtLYK3 autophosphorylation activity** ***in vitro*** **.** The purified intracellular regions of WT MtLYK3, MtLYK3[G334E], and MtLYK3[T480A], fused to the C terminus of GST, were analyzed for their autophosphorylation activity *in vitro* using radiolabeled ATP (γ-^32^P ATP) and phosphorimaging (PI). The coomassie blue staining (CB) shows the protein loading.
(TIF)

Figure S3 **Various (putative) phosphorylation sites are differentially required for MtLYK3 biological activity in** ***Nicotiana*** **.** MtLYK3-sYFP2 mutated variants were co-produced with MtNFP-mCherry fusion in *Nicotiana* leaves: MtLYK3[T285A S286A T300A]+MtNFP (1); MtLYK3[T433A]+MtNFP (2); MtLYK3[T512A]+MtNFP (3); MtLYK3[T480A]+MtNFP (4). Macroscopic observation (left panel) and Evans blue staining (right panel) are depicted 48 hai. Bar is 1 cm.
(TIF)

Table S1 **Primer and linker sequences.**
(DOC)

Materials and Methods S1 (DOC)
