# Supplementary figures and images for: Interaction of Medicago truncatula Lysin Motif Receptor-Like Kinases, NFP and LYK3, Produced in Nicotiana benthamiana Induces Defence-Like Responses
Source: PLoS One. 2013 Jun 4;8(6):e65055. doi: 10.1371/journal.pone.0065055 (PMC3672211; doi:10.1371/journal.pone.0065055)

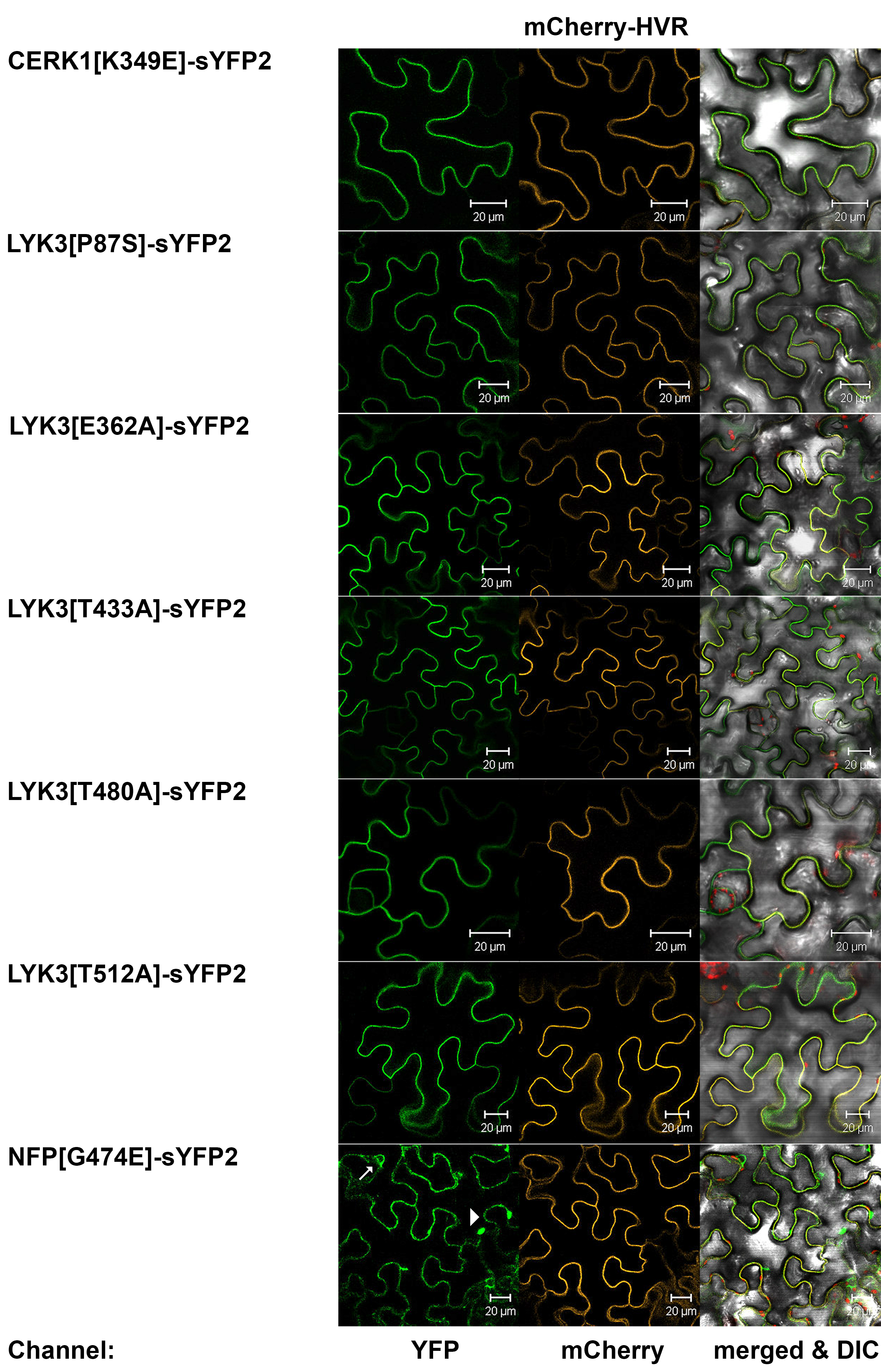

Supplement: Figure S1 — Subcellular localization of various protein fusions in Nicotiana leaf epidermal cells. The plasma membrane marker, mCherry-HVR, was co-produced with the designated fusions in Nicotiana leaf epidermal cells, and the fluorescence (viewed from abaxial side) was imaged 24 hai using confocal laser scanning microscopy. From left to right: green fluorescence of sYFP2; orange fluorescence of mCherry; superimposition of green, orange, and far-red (chlorophyll) fluorescence with the differential interference contrast (DIC) image. Bars are 20 µm. Note 1: in case of subcellular localization of MtNFP[G474E]-sYFP2 fusion, strong fluorescent puncta (indicated with an arrowhead) at the cell boundary of many cells (sometimes in association with nuclei), and pronounced ER localization (indicated with an arrow) of the fusion were still visible at 48 hai. Nevertheless, some cells showed a more uniform pattern of fluorescence at the cell boundary, and this observation, together with a partial insensitivity of this mutated variant to the PNGaseF treatment [22], indicated that at least some MtNFP[G474E] fusion had reached the PM. Note 2: as all kinase-inactive MtLYK3 variants were produced and correctly localized to the plasma membrane in Nicotiana leaf epidermal cells, their lack of biological activity can be attributed to the general abolishment of kinase activity rather than to an individual effect of a particular mutation. (TIF) [file pone.0065055.s001.tif]

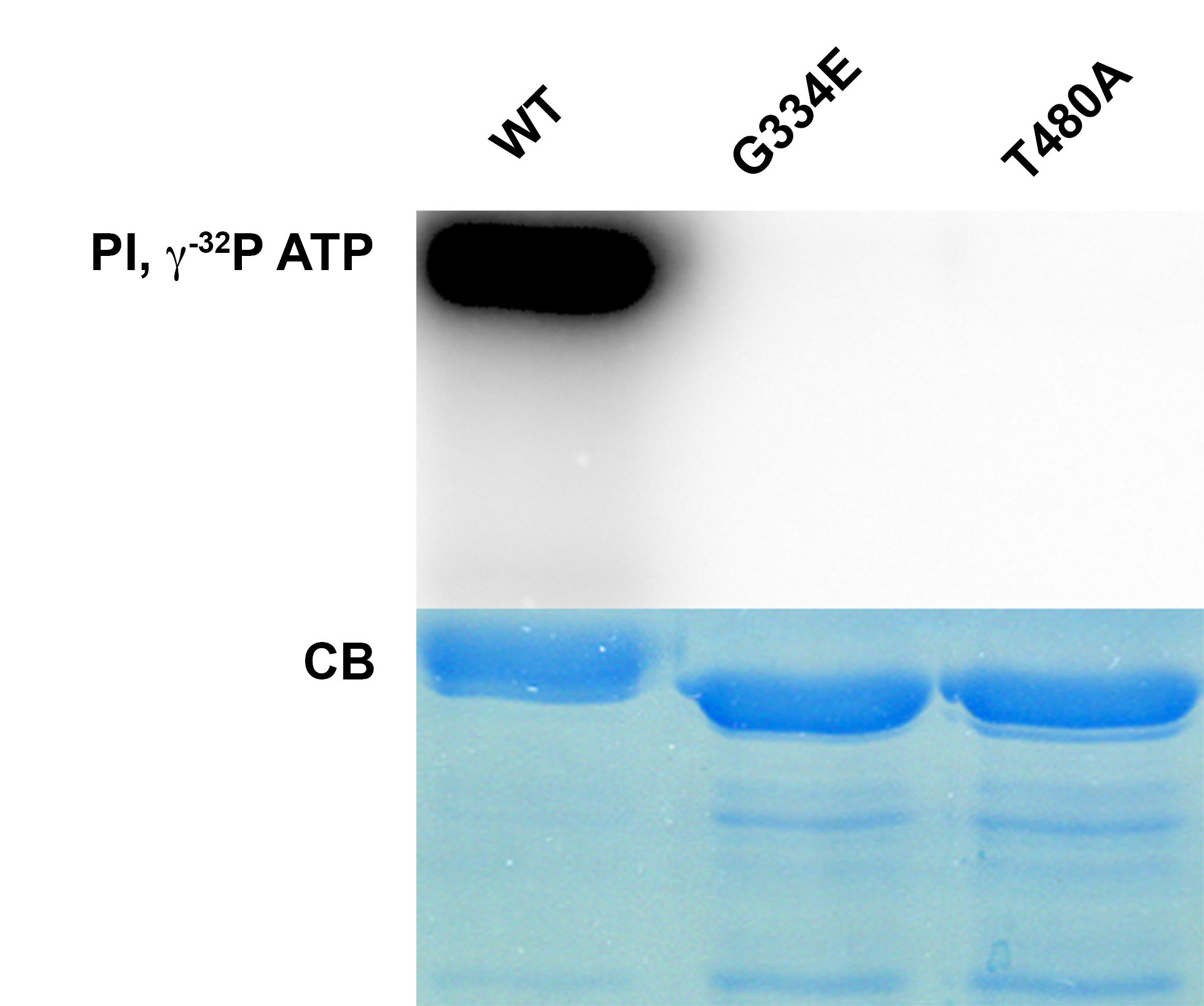

Supplement: Figure S2 — Effect of the Thr 480 Ala substitution on MtLYK3 autophosphorylation activity in vitro . The purified intracellular regions of WT MtLYK3, MtLYK3[G334E], and MtLYK3[T480A], fused to the C terminus of GST, were analyzed for their autophosphorylation activity in vitro using radiolabeled ATP (γ-32P ATP) and phosphorimaging (PI). The coomassie blue staining (CB) shows the protein loading. (TIF) [file pone.0065055.s002.tif]

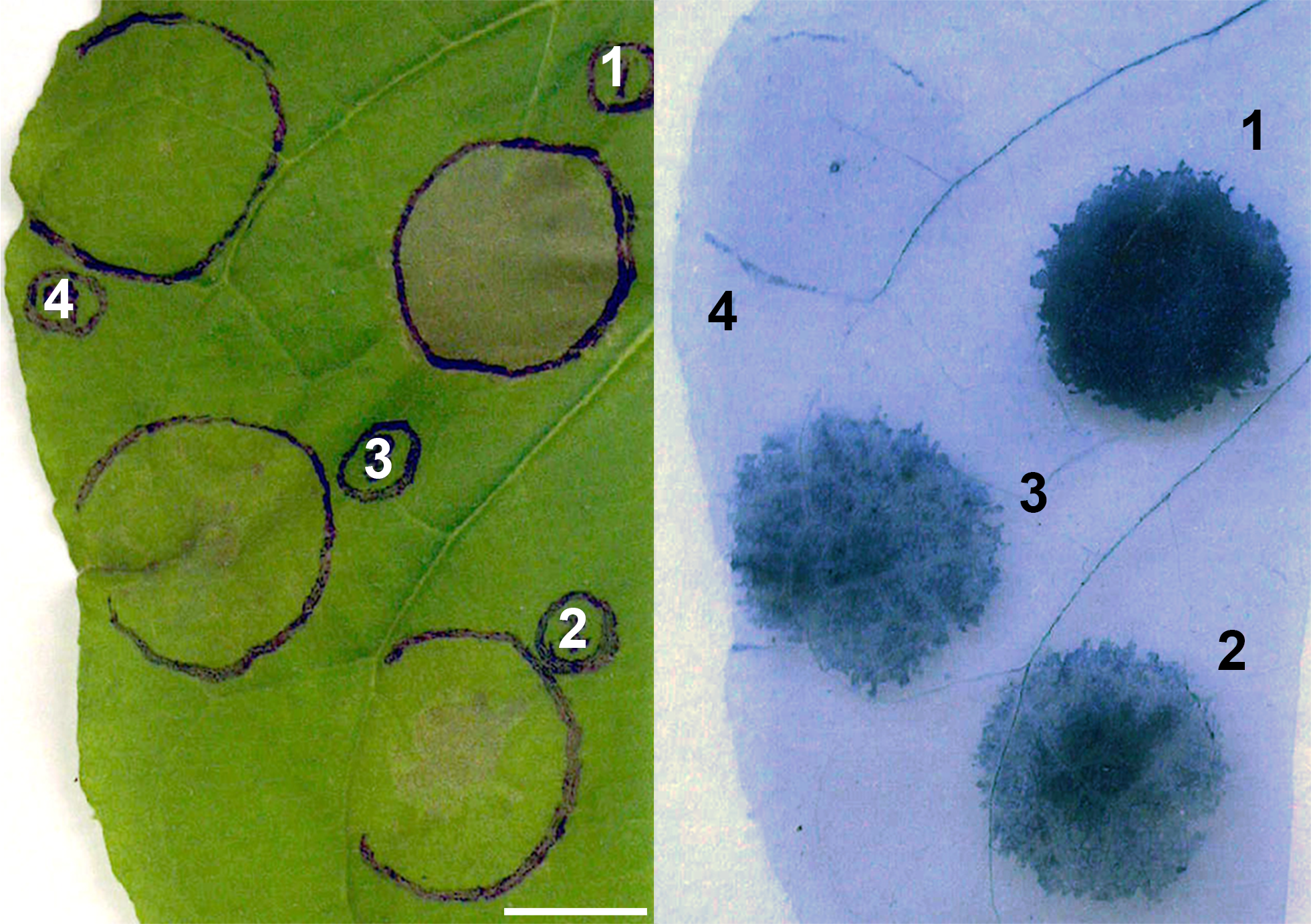

Supplement: Figure S3 — Various (putative) phosphorylation sites are differentially required for MtLYK3 biological activity in Nicotiana . MtLYK3-sYFP2 mutated variants were co-produced with MtNFP-mCherry fusion in Nicotiana leaves: MtLYK3[T285A S286A T300A]+MtNFP (1); MtLYK3[T433A]+MtNFP (2); MtLYK3[T512A]+MtNFP (3); MtLYK3[T480A]+MtNFP (4). Macroscopic observation (left panel) and Evans blue staining (right panel) are depicted 48 hai. Bar is 1 cm. (TIF) [file pone.0065055.s003.tif]
