# Supplementary material for: Interaction of Medicago truncatula Lysin Motif Receptor-Like Kinases, NFP and LYK3, Produced in Nicotiana benthamiana Induces Defence-Like Responses
Source: PLoS One. 2013 Jun 4;8(6):e65055. doi: 10.1371/journal.pone.0065055 (PMC3672211; doi:10.1371/journal.pone.0065055)
Supplement: Table S1 — Primer and linker sequences. (DOC) [file pone.0065055.s004.doc]

Table S1. Primer and linker sequences.

| **Name** | **Type** | **Sequence** |
| --- | --- | --- |
| **MtNFP fw** | Cloning (NheI) | GGGGCTAGCATGTCTGCCTTCTTTCTTC |
| **MtNFP (no stop) rev** | Cloning (EcoRI) | GGGAATTCACGAGCTATTACAGAAGTAA |
| **MtNFP (stop) rev** | Cloning (EcoRI) | GGGAATTCTCAACGAGCTATTACAGAAGTAA |
| **MtLYK3 fw** | Cloning (NheI) | GGGGCTAGCATGAATCTCAAAAATGGATTAC |
| **MtLYK3 (no stop) rev** | Cloning (EcoRI) | GGGAATTCTCTAGTTGACAACAGATTTATG |
| **MtLYK3 (stop) rev** | Cloning (EcoRI) | GGGAATTCTCATCTAGTTGACAACAGATTTATG |
| **AtCERK1 fw** | Cloning (NheI) | GGGGCTAGCATGAAGCTAAAGATTTCTC |
| **AtCERK1 (no stop) rev** | Cloning (EcoRI) | GGGGAATTCCCGGCCGGACATAAGAC |
| **MtDMI2 fw** | Cloning (NheI) | GGGGCTAGCATGATGGAGTTACAAGTTATT |
| **MtDMI2 rev** | Cloning (KpnI) | GGGGGTACCTCTCGGCTGTGGGTGAG |
| **Linker to FP** |  | GAATTC for all the constructs, except for *MtDMI2-sYFP2*: GGTACC |
| **Linker to 3xFlag tag** |  | GAATTCCGGGCTGACTACAAAGACCATGACGGTGATTATAAAGATCATGACATC |
| **NbACRE31 fw** | qRT- PCR | AAGGTCCCGTCTTCGTCGGATCTTCG |
| **NbACRE31 rev** | qRT- PCR | AAGAATTCGGCCATCGTGATCTTGGTC |
| **NbACRE132 fw** | qRT- PCR | AAGGTCCAGCGAAGTCTCTGAGGGTGA |
| **NbACRE132 rev** | qRT- PCR | AAGAATTCCAATCCTAGCTCTGGCTCCTG |
| **NbCYP71D20 fw** | qRT- PCR | AAGGTCCACCGCACCATGTCCTTAGAG |
| **NbCYP71D20 rev** | qRT- PCR | AAGAATTCCTTGCCCCTTGAGTACTTGC |
| **NbHIN1 fw** | qRT- PCR | GAGGGTCACAAGAATACTAGCAGC |
| **NbHIN1 rev** | qRT- PCR | CGCATGTAAAGCTTCACTTCCATCTC |
| **NbPR1a acidic fw** | qRT- PCR | CCTCGTACATTCTCATGGTCAAT |
| **NbPR1a acidic rev** | qRT- PCR | CCATTGTTACACTGAACCCTAGC |
| **NbPR1 basic fw** | qRT- PCR | GTTGCTTGTTTCATTACCTTTGC |
| **NbPR1 basic rev** | qRT- PCR | TTCTCATCGACCCACATTTTTAC |
| **NbEF1 α fw** | qRT- PCR | GCTGCTGCAACAAGATGGATG |
| **NbEF1 α rev** | qRT- PCR | CGAGCATGTTGTCACCTTCCA |

Sequences for restriction sites are underlined.
