# Supplementary material for: Interaction of Medicago truncatula Lysin Motif Receptor-Like Kinases, NFP and LYK3, Produced in Nicotiana benthamiana Induces Defence-Like Responses
Source: PLoS One. 2013 Jun 4;8(6):e65055. doi: 10.1371/journal.pone.0065055 (PMC3672211; doi:10.1371/journal.pone.0065055)
Supplement: Materials and Methods S1 — (DOC) [file pone.0065055.s005.doc]

SUPPORTING INFORMATION MATERIALS & METHODS

**Microscopic analysis**

Was carried as described (20).

**Expression in *Escherichia coli* and kinase activity of the MtLYK3 intracellular region**

Was carried as described (20).
